# Integrating Occupational Health and Safety into Enterprise Risk Management: a structural evaluation

**DOI:** 10.3389/fpubh.2025.1608227

**Published:** 2025-08-04

**Authors:** Yalçın Kılıç, Özalp Vayvay

**Affiliations:** ^1^Institute of Pure and Applied Sciences, Marmara University, Istanbul, Türkiye; ^2^Faculty of Engineering and Natural Sciences, Istanbul Health and Technology University, Istanbul, Türkiye

**Keywords:** Enterprise Risk Management (ERM), Occupational Health and Safety (OHS), internal audit, risk integration, organizational resilience, risk governance, compliance, workplace safety

## Abstract

**Introduction:**

This study aims to investigate the extent to which Occupational Health and Safety (OHS) risks can be incorporated into the broader framework of Enterprise Risk Management (ERM). Although both systems were developed with similar goals—identifying, assessing, and mitigating risks—they have often operated independently. The research explores whether aligning OHS practices with ERM strategies, particularly through internal audit mechanisms, can foster a more unified and efficient approach to organizational risk management.

**Method:**

A qualitative document analysis was conducted, examining current national legislation, international standards such as ISO 31000 (Risk Management) and ISO 45001 (Occupational Health and Safety), and selected academic studies. The evaluation focused on structural similarities, procedural intersections, and the functional roles of personnel involved in ERM, Internal Audit (IA), and OHS processes.

**Results:**

The analysis revealed a substantial convergence between ERM and OHS in terms of risk identification techniques, prevention-based methodologies, and monitoring processes. The responsibilities of internal auditors and occupational safety specialists display notable overlaps, particularly in areas such as compliance, documentation, hazard assessment, and performance reporting. These parallels support the feasibility of integrating OHS risk management into the ERM structure.

**Conclusion:**

For a more effective and holistic approach to enterprise-level risk governance, it is essential to include Occupational Health and Safety risks within the ERM framework. This integration would not only streamline risk management activities but also enhance audit efficiency and organizational resilience. Establishing a closer operational relationship between OHS units and internal audit systems would contribute to safer working environments and more strategic risk oversight.

## 1 Introduction

Risk has long been a defining concept for both individuals and institutions. Initially conceptualized in the 17th century through maritime trade and insurance practices, the notion of risk has since evolved into a multidimensional subject explored across disciplines—from the social sciences to management studies. Today, risk management at the organizational level has expanded beyond financial threats to encompass a broad range of concerns, including operational, strategic, and sustainability-related risks, under the umbrella of Enterprise Risk Management (ERM). Despite this evolution, the integration of domains such as Occupational Health and Safety (OHS) into ERM frameworks remains limited. This study seeks to explore the position of OHS within ERM structures and to build a conceptual bridge between the two systems (1, 2). The early focus was predominantly on financial and operational uncertainties; however, with the globalization of markets and increasing complexity of business processes, the scope of risk management expanded to include environmental, reputational, technological, and regulatory risks. This broader, more strategic approach laid the groundwork for what is now referred to as Enterprise Risk Management (ERM)—a comprehensive framework aimed at identifying, assessing, and mitigating all types of risks that may affect an organization’s objectives (3, 4).

While the formalization of ERM as a concept is relatively recent, the roots of Occupational Health and Safety (OHS) reach back much further in human history. Evidence suggests that even in ancient civilizations, particularly within the domains of health and labor, rudimentary forms of occupational safety practices were observed. For instance, Hippocrates documented lead poisoning among miners in ancient Greece. However, OHS as a systemic and regulated field of practice emerged during the Industrial Revolution in the 19th century, when rapid industrialization led to a surge in workplace accidents and occupational diseases (5). In response to these challenges, legal frameworks and institutional structures began to take shape, aiming to safeguard worker health and prevent occupational hazards. Today, OHS is institutionalized through global standards such as ISO 45001 (6) and national legislation like Turkey’s Occupational Health and Safety Law No. 6331 (7).

Despite their distinct historical origins, both ERM and OHS share a central objective: to identify, control, and mitigate risks that may jeopardize the functioning, safety, and sustainability of an organization. ERM addresses these risks from a holistic, enterprise-wide perspective, incorporating strategic, financial, operational, and compliance-related risks into a unified framework (8, 9). OHS, on the other hand, focuses specifically on workplace-related hazards, including physical, chemical, biological, ergonomic, and psychosocial risks that may affect employee health and safety. Both systems utilize similar methodologies such as risk assessment, hazard identification, preventive controls, performance monitoring, and continuous improvement strategies (10, 11).

Establishing either an ERM or OHS system within an organization is a critical but not sufficient step in ensuring long-term success. Risk identification and control efforts must be supported by robust internal oversight mechanisms. Within the ERM context, internal auditing (IA) plays a pivotal role in evaluating the design, implementation, and effectiveness of risk management activities. The Institute of Internal Auditors (IIA) defines internal auditing as “an independent, objective assurance and consulting activity designed to add value and improve an organization’s operations” [(12), p. 23]. Internal auditors are expected to bring a disciplined and systematic approach to evaluating governance structures, internal controls, and risk processes (13, 14).

Likewise, within the scope of OHS, similar monitoring and audit functions are carried out by OHS professionals, including occupational safety specialists and workplace physicians, whose roles and responsibilities are defined in national regulations such as the Occupational Health and Safety Services Regulation (15). These professionals are tasked with hazard identification, safety training, regulatory compliance, and workplace inspections. Their work ensures not only the implementation of preventive measures but also the long-term sustainability of OHS systems.

Although ERM and OHS were developed as distinct systems and are often managed by separate departments within organizations, their functional similarities are undeniable. Both operate on the basis of continuous risk assessment, utilize data-driven decision-making tools, and aim to protect organizational assets—whether they are physical (e.g., facilities, machines), human (e.g., employees), or reputational. Despite these overlaps, OHS risks are often excluded from ERM frameworks, and consequently, internal audit units rarely address occupational safety issues in their risk portfolios (1, 4). This compartmentalized approach may lead to duplication of efforts, communication gaps, and inefficiencies in overall risk governance.

Given the increasing complexity of organizational risks and the interdependence between employee well-being and operational performance, there is a compelling need to re-evaluate the structural boundaries between ERM and OHS. Scholars have begun to argue for the integration of OHS risks into the broader ERM landscape, emphasizing that such a merger could enhance overall risk visibility, reduce redundancies, and create a more resilient organizational culture (8, 9).

Accordingly, this study seeks to explore whether it is possible—and indeed beneficial—to integrate OHS into ERM structures and whether OHS audits can be incorporated into internal audit functions. The research addresses these questions by conducting a detailed review of legal frameworks, professional guidelines, and academic literature, aiming to establish a conceptual and practical foundation for an integrated risk management model.

The primary aim of this study is to evaluate the feasibility and strategic relevance of integrating Occupational Health and Safety (OHS) into the framework of Enterprise Risk Management (ERM). Specifically, it seeks to determine whether OHS-related risks—which have traditionally been addressed through standalone systems—can be systematically embedded within the ERM process and overseen through internal audit mechanisms. The study also investigates the structural and operational compatibility of OHS units with internal audit departments, focusing on shared methodologies such as risk assessment, monitoring, documentation, and reporting.

While the integration of various risk domains under the ERM umbrella has been widely discussed in the context of financial, operational, reputational, and IT risks (4, 10), the systematic inclusion of OHS risks into ERM remains a largely underexplored area in academic literature. Most studies treat ERM and OHS as distinct systems with separate regulatory frameworks, managerial responsibilities, and audit functions (8, 9). This fragmented approach fails to recognize the growing complexity of workplace environments and the strategic value of holistic risk governance.

This research addresses a critical gap in the literature by bringing together two disciplines that have developed along parallel trajectories but have rarely been examined in an integrated manner. It proposes a conceptual model that situates OHS within ERM, not merely as a compliance-based obligation but as a strategic risk category with implications for organizational sustainability, employee well-being, and governance quality.

Moreover, the study contributes to the theoretical discourse on risk-based internal auditing by suggesting that internal audit units can expand their scope to include OHS audits, thereby fostering a more unified and effective risk oversight system (12, 14). From a practical standpoint, this integrated perspective could assist organizations in eliminating redundant processes, enhancing transparency, and reinforcing a safety-oriented corporate culture. In doing so, the study aligns with contemporary calls for cross-functional risk management and adds a novel, interdisciplinary dimension to the existing literature on enterprise risk, occupational safety, and internal control systems.

## 2 Method

### 2.1 Search strategy

This study employed a qualitative literature review design to examine the possibility and practicality of integrating Occupational Health and Safety (OHS) within Enterprise Risk Management (ERM) frameworks. The search strategy aimed to identify legal texts, international standards, and academic literature that explicitly or implicitly discuss ERM, OHS, internal auditing, and risk integration.

The literature search was conducted using several academic databases, including ScienceDirect, Google Scholar, Web of Science, and Scopus, alongside official websites of international organizations such as the Institute of Internal Auditors (IIA), ISO (International Organization for Standardization), and COSO (Committee of Sponsoring Organizations). Keywords used in the search included: enterprise risk management, internal audit, occupational health and safety, risk integration, risk governance, and safety auditing. Boolean operators such as AND, OR, and NOT were used to refine and expand the search results.

In addition, national regulations from Turkey—such as Occupational Health and Safety Law No. 6331 and the Regulation on Occupational Health and Safety Services (7)—were reviewed as part of the legal framework. A total of 74 documents were included in the final analysis, comprising 28 academic articles, 12 international standards and guidelines (e.g., ISO 31000, ISO 45001), 18 legal or regulatory documents, and 16 professional publications or white papers. A flow diagram summarizing the selection and exclusion process has been included in the Appendix to enhance transparency. Sources were screened in accordance with the inclusion criteria and independently cross-checked by both authors to ensure comprehensiveness and relevance.

The inclusion criteria focused on publications that:

- Discussed ERM and OHS as individual or intersecting systems;
- Included definitions, responsibilities, or audit mechanisms related to internal control or OHS;
- Were published between 2000 and 2023;
- Were available in English or Turkish.

Documents that lacked peer review, were overly descriptive without methodological grounding, or did not clearly address organizational-level risk management were excluded.

### 2.2 Data extraction

After identifying and screening relevant sources, the data extraction phase focused on isolating content related to four thematic areas: Conceptual frameworks of ERM and OHS; Roles and responsibilities of internal auditors and OHS specialists; Legal and procedural standards, including ISO 31000, ISO 45001, COSO ERM Framework, and Turkish OHS legislation; Audit mechanisms, including scope, frequency, methodology, and integration potential between IA and OHS functions.

Information from each source was recorded in a structured matrix to track the origin, year of publication, type of document (e.g., academic article, standard, regulation), thematic relevance, and key findings. The goal was to ensure systematic comparison across different perspectives, especially between normative standards and practical implementation reports. Data extraction and preliminary theme development were jointly conducted by both authors to strengthen analytical reliability. Discrepancies in thematic coding were resolved through discussion until full consensus was achieved. Additionally, document types were classified to differentiate peer-reviewed sources from regulatory or institutional texts, as quality appraisal was not uniformly applicable across all source types.

Wherever possible, direct citations and quotations were recorded for reference accuracy and to support argumentation in the discussion section.

### 2.3 Data analysis

The extracted data were analyzed using qualitative content analysis, with an inductive coding approach to identify recurring patterns, conceptual overlaps, and contradictions between OHS and ERM systems. The analysis began with open coding, in which key terms such as “risk control,” “audit function,” “preventive strategy,” and “organizational sustainability” were tracked across sources.

Codes were then grouped into broader categories reflecting: Structural similarities between ERM and OHS; Operational and procedural alignment; Gaps in risk governance due to their separation; the potential benefits and challenges of integration. Although intercoder reliability metrics (e.g., Cohen’s Kappa) were not calculated due to the nature of the analysis, the involvement of both authors in the coding and interpretation process served as an internal validation measure. An example of how data were coded into themes is provided in Table 1, illustrating how references to “preventive safety actions” were classified under “Proactive Risk Management.”

**Table 1:** Example of data coding and theme assignment.

Source type	Extracted quote or concept	Initial code	Final theme
ISO 45001 (2018)	“Organizations shall identify hazards and assess risks.”	Hazard identification	Risk Identification & Control
Academic Article	“OHS must be aligned with strategic objectives.”	Strategic alignment	Integration with ERM Cycle
COSO Framework	“Risk responses should be preventive or detective.”	Preventive approach	Proactive Risk Management
Turkish Law No. 6331	“Employers are obliged to take necessary safety measures.”	Legal compliance duties	Governance Responsibilities

This table illustrates how specific references and excerpts from the reviewed documents were initially coded and subsequently grouped into broader analytical themes during the content analysis phase.

Through this thematic synthesis, the study aimed to construct a conceptual model illustrating how OHS risks could be incorporated into the ERM cycle and how internal audit mechanisms could accommodate occupational safety assessments.

Triangulation was ensured by consulting multiple types of documents (e.g., legal texts, standards, empirical studies), and findings were interpreted with reference to the International Professional Practices Framework (IPPF) (15) and ISO 31000 guidelines (10).

## 3 Results

### 3.1 Comparison of ERM and OHS systems

Both Enterprise Risk Management (ERM) and Occupational Health and Safety (OHS) are structured systems aimed at identifying, analyzing, and mitigating organizational risks. While ERM targets strategic, financial, operational, and compliance-related uncertainties, OHS focuses specifically on hazards affecting employee health and workplace safety. Despite differing focal points, the overarching goal in both systems is to reduce risk exposure and enhance sustainability.

As illustrated in Table 2, both frameworks emphasize enterprise-wide applicability and are grounded in the principle of safeguarding people and assets. ISO 31000:2009 and Turkey’s Law No. 6331 clearly state that their scope covers all sectors and all sizes of organizations, reinforcing the universality of their application.

**Table 2:** Comparison of ERM and OHS in terms of purpose and scope.

Feature	ERM	OHS
Primary focus	Strategic, Financial, Operational, Compliance	Physical, Chemical, Biological Workplace Risks
Legal/standard reference	ISO 31000	Law No. 6331, ISO 45001
Applicability	All sectors and organizational levels	All employers and employees
Risk target	Organization-wide objectives	Employee health and workplace safety

According to Table 2, both systems adopt a holistic risk governance mindset, and their scopes significantly overlap in terms of enterprise coverage and human-centered objectives.

The definition of risk has evolved in both domains. ERM describes risk as any uncertainty that affects organizational objectives, whether positively or negatively. OHS definitions remain more conservative, focusing on potential harm resulting from hazards. However, as seen in Table 3, both systems engage in systematic risk assessment, prioritization, and control.

**Table 3:** Definitions and approaches to risk in ERM and OHS.

Criterion	ERM	OHS
Risk definition	Effect of uncertainty on objectives (ISO 31000)	Probability of harm arising from workplace hazards
Risk assessment	Based on impact and likelihood, linked to objectives	Grading risks to prioritize control and mitigation
Risk appetite	Defined by top management	Legally restricted – no tolerance for unaddressed risks
Goal	Reasonable assurance	Acceptable risk levels via mandatory controls

As Table 3 demonstrates, while ERM allows for a degree of risk tolerance based on institutional appetite, OHS follows a stricter, compliance-driven path, requiring all risks to be addressed through corrective and preventive actions.

Despite differences in terminology and frameworks, the implementation procedures of ERM and OHS reveal striking parallels. Both systems begin with risk identification, followed by evaluation, control planning, documentation, and continuous monitoring. The COSO framework in ERM and ISO 45001 in OHS each emphasize iterative improvement and feedback loops (Table 4).

**Table 4:** Comparison of implementation steps in ERM and OHS.

Implementation stage	ERM (COSO Framework)	OHS (ISO 45001/Law No. 6331)
Risk identification	Event identification	Hazard identification
Risk analysis	Likelihood-impact matrix	Probability-severity analysis
Risk control	Avoid, reduce, share, accept	Eliminate, reduce, isolate, PPE
Monitoring	Internal audit, continuous review	Field supervision, OHS board inspections
Documentation	Risk register, audit reports	Risk assessment forms, control measure records

As outlined in Table 4, both systems rely heavily on documentation and accountability, and both aim to embed risk control into daily operations.

Responsibility for risk management in ERM lies primarily with senior management and the board of directors, while in OHS the employer bears non-transferable legal responsibility. Still, both systems involve the whole organization in a layered responsibility structure, as presented in Table 5.

**Table 5:** Responsibility structures in ERM and OHS systems.

Role level	ERM responsibilities	OHS responsibilities
Senior management	Set risk appetite, approve controls	Ensure all legal obligations are fulfilled
Risk/OHS committees	Coordinate risk functions	OHS Board decisions and strategy implementation
Operational units	Implement risk controls	Apply workplace-specific safety measures
All employees	Report incidents, comply with procedures	Follow safety protocols, participate in training

Table 5 highlights the hierarchical but integrated governance approach shared by both systems, reinforcing that risk responsibility is collective, though ultimately accountable to top leadership.

### 3.2 Audit structures in ERM and OHS

ERM systems rely on internal audit (IA) units to monitor and evaluate risk activities. OHS systems, similarly, are monitored by occupational safety specialists. Table 6 summarizes the personnel and reporting structures.

**Table 6:** Internal organizational roles and reporting lines.

Unit	Responsible personnel	Reports to
Internal audit	Internal Auditor	Executive Board
OHS unit	Occupational Safety Specialist	Employer (OHS Board)

According to Table 6, both roles operate within formalized structures and report to the highest organizational authority, ensuring independence and oversight.

Both ERM and OHS systems are subject to external audits. In ERM, this is done via independent auditors. In OHS, it is overseen by labor inspectors. These structures are compared in Table 7.

**Table 7:** Comparison of internal and external audit mechanisms.

Feature	Internal audit	Occupational safety	External oversight
Appointed by	Internal management or outsourced	Employer or OHS service provider	State labor authority
Objective	Efficiency, risk assurance	Workplace safety compliance	Legal compliance
Frequency	Ongoing	Regular inspections	Scheduled or triggered
Authority	Advisory	Advisory	Enforcement, sanctions

Table 7 demonstrates that while internal audits are advisory in nature, external audits carry enforcement authority, providing a dual layer of risk governance.

Both internal auditors and OHS professionals are bound by strict ethical and independence standards. As illustrated in Table 8, they must operate free from undue influence to ensure objective reporting.

**Table 8:** Ethical principles and independence requirements.

Criteria	Internal auditor	Occupational safety specialist
Ethical obligations	Objectivity, independence, confidentiality	Professional neutrality, legal protection
Legal protection	No extra assignments allowed	Cannot be dismissed due to reporting
Professional boundaries	No administrative authority	Advisory role only

Table 8 confirms the requirement for both professions to remain independent in their assessment roles, supported by legal frameworks ensuring their protection.

Internal auditors and OHS experts must be certified and trained in their fields, but due to the complexity of organizations, collaboration with specialists is often necessary. This is reflected in Table 9.

**Table 9:** Professional competency and advisory scope.

Feature	Internal auditor	Occupational safety expert
Certification required	Yes (IIA or equivalent)	Yes (Ministry-certified)
Advisory scope	Fraud, IT, operations, finance	Electrical, mechanical, chemical safety
Limits of knowledge	May require external consultancy	External expertise often necessary

According to Table 9, both professionals are expected to operate across a broad range of issues, often requiring multidisciplinary collaboration to ensure effectiveness.

**Figure 1:**
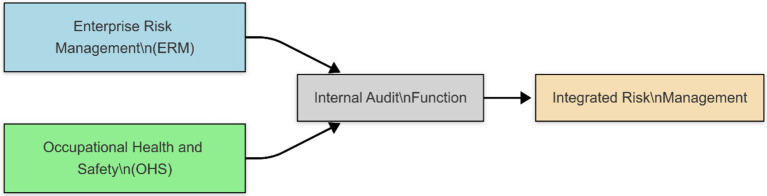
Integration of Occupational Health and Safety into Enterprise Risk Management.

This conceptual model illustrates the structural pathway through which Occupational Health and Safety (OHS) practices can be integrated into the broader Enterprise Risk Management (ERM) framework. As shown, both ERM and OHS systems function as distinct yet complementary risk control mechanisms. Each independently identifies and mitigates specific organizational risks. These systems converge through the Internal Audit function, which serves as a central oversight mechanism. Through this convergence, a more unified and comprehensive Integrated Risk Management system is formed, where OHS is no longer treated as a siloed activity but becomes an essential component of overall enterprise risk governance. This model highlights the feasibility of such integration and underscores the pivotal role of internal audit in facilitating cross-functional risk oversight (Figure 1).

## 4 Discussion

Occupational Health and Safety (OHS) has historically centered on protecting human life—an ethical imperative that remains uncontested across all organizational settings. In recent years, however, OHS has evolved beyond health preservation to support broader business sustainability goals. These include safeguarding physical infrastructure, financial assets, production continuity, and corporate reputation (5). OHS, therefore, should not be viewed solely as a regulatory requirement. Instead, it must be recognized as a strategic pillar essential for ensuring enterprise continuity and organizational resilience.

The development of Enterprise Risk Management (ERM), as reflected in frameworks like COSO (3, 4), is based on the principle of addressing all types of risks holistically. ERM acknowledges the interconnectedness of risks, where a failure in one business unit can trigger cascading effects across the organization. For instance, a financial shortfall may lead to reputational damage, legal consequences, or operational disruptions.

OHS risks demonstrate this same interconnected impact. A workplace injury, for example, can halt production, delay supply chains, incur legal liabilities, and damage public trust. Such consequences illustrate that OHS risks are not peripheral but core strategic risks. Yet, many ERM models and international standards overlook this, or refer to OHS only briefly under operational risks (10). This limited treatment weakens the holistic intent of ERM frameworks. As the findings of this study show, OHS and ERM share several structural and procedural similarities: both rely on risk identification, documentation, corrective actions, and performance monitoring. Nevertheless, they are often implemented through separate organizational channels, resulting in fragmented oversight and inefficiencies in reporting. In many organizations, risk reports are submitted to top management from different departments and at different times. This disjointed approach reduces the potential for comprehensive, enterprise-level decision-making.

Moreover, categorizing OHS solely as an operational risk is overly restrictive. OHS influences nearly every function of the organization—including HR, logistics, finance, and communications. For this reason, the study proposes that OHS should be elevated to a distinct risk category within ERM, rather than subsumed under operations. The proposed model advocates for the integration of OHS into ERM via a shared Internal Audit (IA) structure. Such integration could promote centralized oversight, consistent reporting, and stronger alignment between safety and enterprise-wide risk objectives.

The role of internal audit in this integration deserves special attention. Originally designed to detect financial fraud, internal auditing has since evolved into a broader assurance and consultancy function. Today, IA supports ERM by identifying vulnerabilities and recommending improvements (12, 14).

Risk-based internal audits now cover diverse areas—such as IT, procurement, and logistics—reflecting the multifaceted nature of enterprise risk. Given this expansion, excluding OHS from IA’s scope represents a missed opportunity. As highlighted in the findings, internal auditors and OHS professionals have overlapping competencies and legal responsibilities. Both act under ethical codes that emphasize objectivity, confidentiality, and independence (ISO 19011:2018; OHS Law No. 6331).

Despite these shared foundations, OHS units and IA departments frequently operate in silos. This lack of coordination results in OHS risks being underrepresented in strategic risk assessments. Internal auditors may lack the technical knowledge to fully address safety risks, while OHS experts—operating outside of IA—struggle to ensure visibility for their findings at the executive level.

Integrating OHS into IA structures would not only address this fragmentation but also offer strategic benefits. These include consolidated reporting, reduced duplication of effort, and a unified approach to risk governance. Under this model, OHS professionals would continue performing their core duties—hazard identification, workplace assessments, and regulatory compliance—but their findings would be incorporated into internal audit reports.

This would ensure that safety-related risks are consistently communicated to top management alongside financial, strategic, and operational risks.

Concerns regarding the independence of OHS reporting under an IA structure are valid but manageable. Internal audit units are structurally designed to operate independently and report directly to audit committees or boards. Housing the OHS function within, or in close coordination with, IA may in fact enhance its institutional autonomy.

Currently, OHS departments often report through operational units like HR or production, which may limit their visibility and influence. A closer alignment with IA would elevate the strategic standing of safety in the organizational hierarchy.

Additionally, both internal auditors and OHS professionals are protected by legal frameworks that ensure their freedom to report findings objectively and without retaliation (OHS Law Art. 8; Regulation 2006 Art. 27). Therefore, structural integration would not compromise professional independence. On the contrary, it could empower safety experts and enhance the speed and clarity of risk communication.

In conclusion, this study highlights the untapped potential of formally integrating OHS into both ERM and IA structures. The conceptual parallels, procedural overlaps, and legal justifications all support such integration. The proposed model offers a remedy to fragmented risk oversight and enhances coherence in organizational governance. For enterprises committed to resilient and ethical risk management, treating OHS as a formal risk category within ERM—backed by internal audit—is not just beneficial; it is essential.

## 5 Conclusion

This study set out to examine whether Occupational Health and Safety (OHS) risks—traditionally managed as a separate domain—can and should be structurally integrated into the framework of Enterprise Risk Management (ERM). The findings from document analysis, regulatory review, and conceptual comparison reveal a significant convergence between OHS and ERM systems in terms of their risk management methodologies, monitoring processes, and assurance mechanisms. Despite this alignment, OHS risks remain largely excluded from formal ERM structures and internal audit functions, resulting in fragmented governance and missed opportunities for organizational resilience.

By evaluating the legal, procedural, and operational dimensions of both ERM and OHS, the study identifies a clear rationale for integration. OHS risks are not limited to compliance or employee welfare; they directly affect strategic goals, operational continuity, financial performance, and corporate reputation. Ignoring them in the ERM cycle undermines the holistic nature of enterprise risk governance. Similarly, the exclusion of OHS activities from internal audit (IA) systems weakens the coherence and effectiveness of enterprise-level risk oversight.

The study argues that OHS should be explicitly recognized as a core risk domain within ERM frameworks and that the activities of occupational safety experts should be coordinated with internal audit functions. This structural alignment would ensure that OHS risks are reported to top management alongside other critical risks, using unified tools and language. Moreover, it would enhance the independence, visibility, and strategic contribution of OHS professionals.

Contrary to concerns about compromised independence, the study suggests that positioning OHS under internal audit may, in fact, strengthen the autonomy and institutional legitimacy of the function. Given that internal audit is already structured to operate independently from business operations, such integration can provide OHS personnel with greater access to decision-makers and ensure that their risk assessments carry appropriate weight in strategic planning.

In conclusion, the integration of OHS into ERM and IA structures is both a practical necessity and a strategic opportunity. It aligns with international standards, promotes a unified approach to risk management, reduces operational silos, and supports the creation of safer, more resilient organizations. For enterprises committed to comprehensive governance and sustainable performance, incorporating OHS risks into the ERM framework is not only advisable—it is imperative.

## Author’s note

This article was produced from the PhD thesis titled “*Internal Audits and Establishment of an Exemplary Model in Terms of Occupational Health and Safety within the Scope of Enterprise Risk Management in Fair Organizations, which are a Link of the Supply Chain*,” prepared by the first author under the supervision of the second author.

## Data Availability

The raw data supporting the conclusions of this article will be made available by the authors, without undue reservation.
